# Clinical benefit and radiological response with BRAF inhibitor in a patient with recurrent ameloblastoma harboring V600E mutation

**DOI:** 10.1186/s12885-018-4802-y

**Published:** 2018-09-12

**Authors:** Gustavo S. Fernandes, Daniel M. Girardi, João Paulo G. Bernardes, Felipe P. Fonseca, Eduardo R. Fregnani

**Affiliations:** 1 Department of Oncology, Hospital Sírio Libanês, SGAS 613, conjunto E lote 95, Asa Sul, Brasília, DF 70200-001 Brazil; 2 Department of Radiology, Hospital Sírio Libanês, São Paulo, Brazil; 3 Department of Oral Surgery and Pathology, School of Dentistry, Universidade Federal de Minas Gerais, Belo Horizonte, Brazil; 4 Oral Medicine Department, Sírio-Libanês Hospital, São Paulo, Brazil

**Keywords:** Ameloblastoma, BRAF, MAPK, Targeted therapy

## Abstract

**Background:**

Ameloblastoma is a slow-growing neoplasm of the jaw, for which the standard treatment is surgical removal of the lesion with high recurrence rates and elevated morbidity. Systemic therapy is not established in the literature.

**Case presentation:**

We present a case of a 29-year-old woman diagnosed with an ameloblastoma of the left mandible who had been subjected to several surgical procedures over twenty years due to multiple local recurrences. Molecular testing revealed a BRAF V600E mutation, and vemurafenib was started. She experienced complete resolution of symptoms related to the disease, and image scans evidenced continuous shrinkage of the neoplastic lesion after eleven months of therapy.

**Conclusion:**

This is the first report showing clinical benefit and radiological response with vemrafenib for recurrent ameloblastoma. Targeted therapy addressing BRAF V600E mutation has the potential to change clinical practice of this rare disease.

## Background

Ameloblastoma is a locally invasive, slow-growing odontogenic neoplasm arising in the jaw, which accounts for 13–58% of odontogenic tumors [1]. The standard treatment is surgery, which can be either conservative (enucleation or curettage) or radical. The first is associated with high recurrence rates (up to 90%), and the latter with significant morbidity [1].

Recently, elucidations of the molecular pathways that lead to development of the disease have revealed new treatment possibilities. By 2014, several papers had reported alterations in the mitogen-activated protein kinase (MAPK) cascade, and the activating mutation BRAF V600E was found in 40–80% of cases [2–5]. Mutations in the Hedgehog pathway were also described and are the second most common genetic alteration. Interestingly, the molecular profiles are correlated with phenotypic features and may also be related to prognosis [5].

These intriguing data led to the assumption that targeted therapy against these pathways may be clinical useful. Indeed, two studies have demonstrated in vitro sensitivity of BRAF inhibitors in ameloblastoma cells harboring BRAF V600E mutation [3, 5], and three other studies have reported successful cases of patients with BRAF V600E mutation treated with BRAF inhibitors [6–8]. Our case report describe another successful case of recurrent ameloblastoma treated with targeted therapy.

## Case presentation

This case describes a 29-year-old woman who was first diagnosed with ameloblastoma as a child at 7 years old. The lesion originated in the ascending branch of the left mandible, and the first surgical procedure was performed in March 1997 followed by disease recurrence in April 1999. A second resection was performed in May 1999, and during the next 16 years, the patient underwent several surgical approaches that were consistently followed by disease recurrence. Some of the procedures were conservative surgeries, but others were radical procedures that left her with several deforming scars. She presented to our clinic in January 2015 with a new magnetic resonance imaging (MRI) that evidenced a right, triangular aspect, paracellarlesion, extending to the homolateral cavernous sinus (13 × 9 mm), which was suspected to be a residual lesion that would have achieved the cavernous sinus by contiguity growth after several surgeries. Her last surgery had been performed in April 2014 and was followed by local radiotherapy in May 2014. She was asymptomatic and not willing to undergo a new invasive procedure. She decided to be followed without further intervention.

**Fig. 1:**
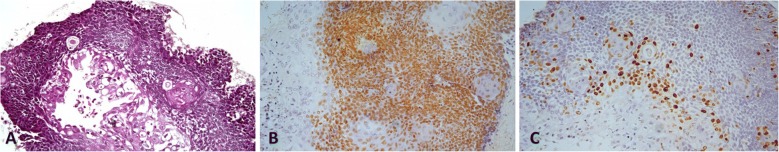
Microscopic and immunohistochemical findings of the tumor. **a** Lossely-arranged central cells and hypercromatic peripheral cells (H&E; 200X). **b** Immunohistochemical staining for p63 (DAB; 200X). **c** Immunohistochemical staining for Ki67 predominantly staining cells located in the peripheral layer

For the next 18 months, she was clinically stable and asymptomatic, but she returned in July 2016 with intense pain on the right side of her face that required multiple hospital visits for intravenous analgesia. MRI revealed an extensive heterogeneous lesion with contrast enhancement centered on the right cavernous sinus anterior to the cavus of Meckel and exhibiting anterior extension towards the upper orbital fissure (measuring approximately 19 × 15 × 16 mm). To identify new treatment possibilities, we decided to perform a new biopsy and conduct molecular testing (Fig. 1). A BRAF mutational analysis by the allele-specific protein chain reaction (PCR) certified test revealed the presence of a BRAF c.1799 T > A;p.V600E mutation corresponding to a V600E amino acid substitution. After tumor board discussion and a careful conversation with the patient, she decided to undergo BRAF inhibitor therapy.

**Fig. 2:**
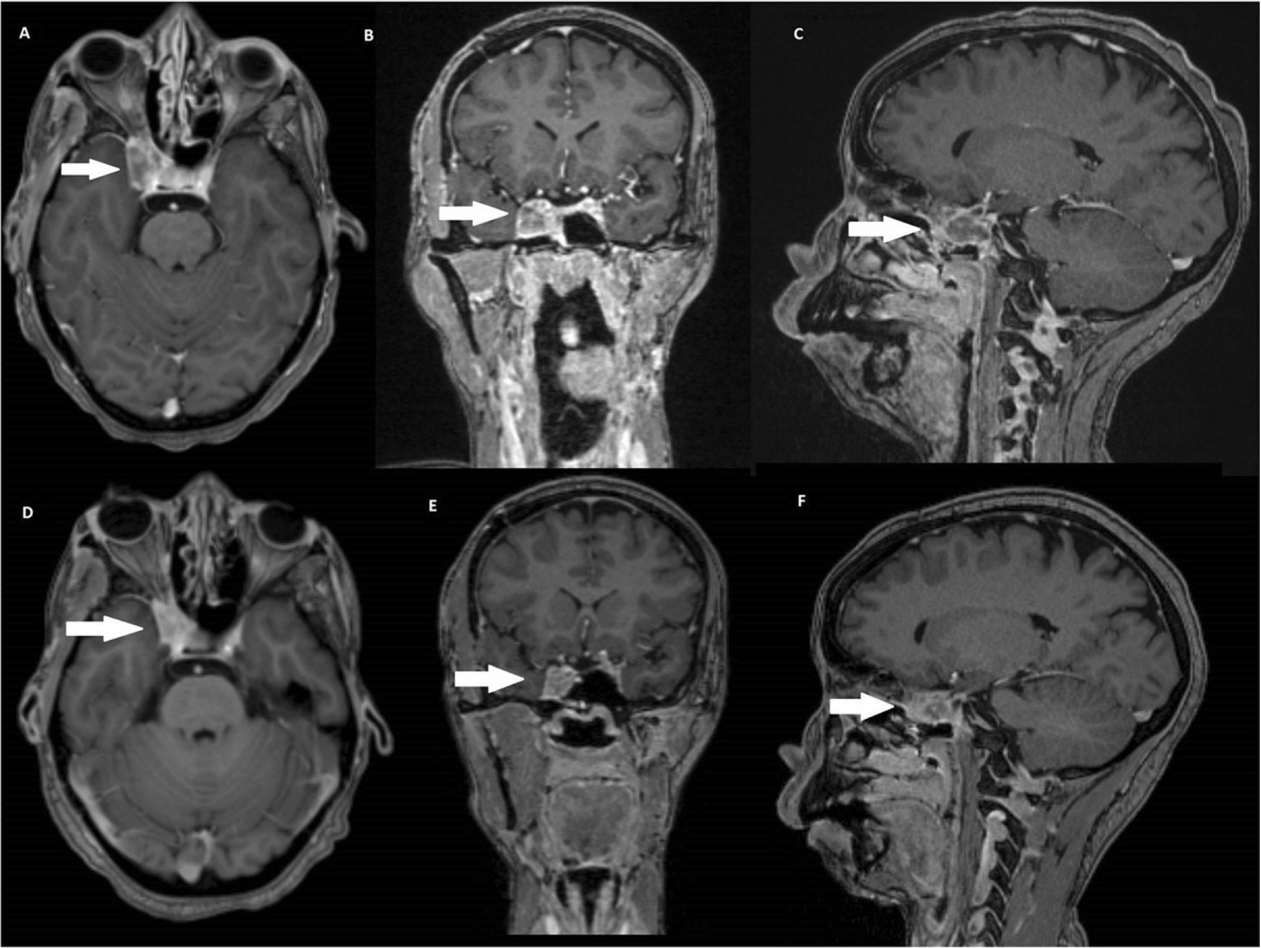
Images **a**, **b**, and **c**: Contrast-enhanced T1-weighted magnetic ressonance imaging (MRI) acquired prior to initiation of treatment (September, 2016), demonstrating lesion with heterogeneous enhancement (arrows) in the right cavernous sinus with insinuation in the superior orbital fissure measuring 24 × 21 × 19 mm. Images **d**, **e** and **+**: Brain MRI one year after the initiation of Vemurafenib (September 2017) showing significant reduction of lesion dimensions, measuring 18 × 13 × 14 mm

A treatment regimen with vemurafenib 960 mg PO twice daily was started on October 4, 2016. Prior to the initiation of therapy, a new MRI performed on September 24 revealed a lesion measuring 24 × 21 × 19 mm. After 2 weeks of therapy, the patient was asymptomatic and was not using any analgesic medication. During the course of therapy, she experienced grade one anorexia, nausea and fatigue, without any severe therapy-related adverse events. MRI performed in April 2017 revealed stable disease (24 × 18 × 15 mm), and her last MRI performed in September 2017 evidenced a reduction of the lesion size (18 × 13 × 14 mm) (Fig. 2). The patient currently remains asymptomatic with excellent tolerance to the medication.

## Discussion and conclusions

Here we report a case of disease control with BRAF inhibitor monotherapy for recurrent ameloblastoma. We prefer monotherapy to dual therapy with an MEK inhibitor anticipating difficulty in obtaining approval from patient’s health insurance, the costs associated with the combination and the lack of data in the literature. The patient experienced clinical benefit with resolution of her symptoms, and image scans have showed progressive shrinkage of lesion dimensions. Ameloblastoma is a rare disease, for which the standard treatment is surgical removal of the tumor [1]. Surgical procedures are associated with high recurrence rates, especially conservative surgeries, and face deformity. Therefore, new treatment options are needed to control the disease in patients experiencing multiple recurrence episodes or who are not suitable for surgical treatment.

In the age of precision medicine, the characterization of molecular pathways that lead to tumorigenesis is essential to develop and clinically test targeted agents that are capable of interfering with crucial molecules involved in cancer development and progression. MAPK is a complex pathway involved in carcinogenesis. This cascade can be activated by fibroblast growth factor receptor 2 (FGFR2) and epithelial growth factor receptor (EGFR), which lead to the activation of downstream RAS, RAF, MEK and ERK. The BRAF V600E mutation leads to constitutive activation of the BRAF protein and is well known to be involved in the carcinogenesis of other histologies, such as melanoma and colorectal cancer [9]. The Hedgehog pathway also plays an important role in tooth development and in the carcinogenesis of ameloblastoma [10, 11]. Mutations in SMO (a Hedgehog signal transduction component) are the second most prevalent somatic mutation and tend to be mutually exclusive with BRAF mutations [5, 12].

In 2014, alterations in the MAPK pathway, especially BRAF mutations, were described by different authors for ameloblastoma tumors. Kurppa et al. reported BRAF V600E mutations in 63% of their samples [2]. Sweeney et al. found the BRAF V600E mutation in 46% of their cases and also described mutations in other genes, such as KRAS, FGFR2, and SMO [5]. Brown et al. showed mutations in several genes, such as BRAF, KRAS, NRAS, FGFR2, SMO, SMARCB1, CTNNB1, and PIK3CA. BRAF V600E was the most common mutation found in 62% of cases [3]. Another study published by Diniz et al. in 2015 showed that the BRAF V600E mutation was present in 82% of cases [4]. A recent study analysed 62 patients with ameloblastoma. Mutations were identified in 57 of these patients (92%) and BRAF V600E was the most prevalent, detected in 60% of patients, followed by SMO mutations identified in 14% of patients [12].

The molecular characteristics of ameloblastoma also seem to be correlated with clinicopathological features. Tumors harboring the BRAF mutation seem to occur more frequently in the mandible and younger patients, whereas SMO mutations are more associated with tumors arising in maxillary of older patients [3, 12–14]. This is in accordance with our case report, in which a young woman was affected with ameloblastoma arising in the ascending ramus of the left side of the mandible. However, after multiple recurrences, the lesion involved the cavernous sinus. We hypothesize that the tumor achieved this anatomical location due to contiguity growth after many surgeries, as described previously in literature [15].

Data concerning the aggressiveness of disease harboring the BRAF mutation are conflicting. Some studies have observed a higher disease-free survival (DFS) in those harboring the BRAF mutation in comparison to BRAF wild-type tumors [3, 5], while another study observed a more aggressive disease with poor DFS for BRAF-V600E mutation tumors [16]. Interestingly, some studies have reported that the risk of recurrence was lower in patients with BRAF-V600E mutation compared with patients harboring more than one gene mutation and with patients harboring SMO mutation [12, 14].

Data supporting the clinical benefit of BRAF inhibitors for patients with ameloblastoma harboring the BRAF mutation are very scarce. Two studies have reported in vitro sensitivity of vemurafenib for ameloblastoma cell lines harboring V600E mutations [3, 5]. Clinical activity has been described in three case reports. Kaye et al. reported a case of ameloblastoma with multiple recurrences after radical surgeries that developed lung metastasis. BRAF V600E was detected, and therapy with dabrafenib (BRAF inhibitor) and trametinib (MEK inhibitor) was started with complete resolution of symptoms and an excellent radiological response [6]. Tan et al. reported a case of recurrent ameloblastoma after a conservative procedure with administration of dabrafenib. The patient experienced an impressive tumor reduction and became eligible for subsequent radical resection of the remaining lesion [7]. Finally, Faden et al. reported a case of an 83-year-old woman with recurrent ameloblastoma harboring the BRAF V600E mutation who was not suitable for further surgical treatment due to comorbidities. She received dabrafenib and experienced an impressive reduction of the lesion size and a sustained response after 12 months of therapy [8].

Despite being very preliminary data, these findings suggest that the molecular features of ameloblastoma could be useful for the selection of targeted therapy. To the best of our knowledge, we have described the first case of ameloblastoma with BRAF V600E that experienced clinical benefit and a radiological response with vemurafenib. Further prospective studies addressing the hole of BRAF and BRAF/MEK inhibition are needed to clarify the best treatment regimen for these patients, and larger molecular studies are warranted to clarify the roles of other mutations outside the MAPK cascade, such as the SMO mutation involved in the Hedgehog cascade [3, 5].

## Abbreviations

DFS: Disease-free survival
EGFR: Epithelial growth factor receptor
FGFR2: Fibroblast growth factor receptor 2
MAPK: Mitogen-activated protein kinase
MRI: Magnetic resonance imaging.
PCR: Protein chain reaction.
